# Tobacco use and psychotic experiences : A cross sectional study among the general population

**DOI:** 10.1192/j.eurpsy.2026.11854

**Published:** 2026-07-31

**Authors:** L. S. Chaibi, M. Khamassi, K. Maagli, M. Cheour, F. Fekih-Romdhane

**Affiliations:** 1 Faculty of Medecine of Tunis; 2 Psychiatry, Razi Hospital, Tunis, Tunisia

## Abstract

**Introduction:**

Tobacco use has been increasingly recognized as a potential risk factor for various mental health issues, including psychotic experiences (PEs). While the relationship between smoking and psychosis has been explored in clinical populations, less is known about this association in the general population, particularly among young adults.

**Objectives:**

This study aimed to investigate the relationship between tobacco use and PEs in a large sample of young adults from the general population across twelve Arab countries.

**Methods:**

A cross-sectional study was conducted from February 1st to April 30, 2024, involving 7,646 participants aged 18 to 35 years. Data were collected using a self-administered online questionnaire, which included the Prodromal Questionnaire-Brief (PQ-B) to assess psychotic experiences and questions on tobacco use. Statistical analyses were performed to examine the association between tobacco use and PEs, adjusting for sociodemographic variables.

**Results:**

The study found that smokers had significantly higher mean PQ-B scores compared to non-smokers (p = .012), indicating a greater prevalence of psychotic experiences among tobacco users. This association remained significant even after controlling for other sociodemographic factors such as age, sex, and education level.

**Conclusions:**

This study highlights a significant association between tobacco use and psychotic experiences among young adults in the general population. The results underscore the importance of addressing tobacco use in mental health interventions, particularly in populations at risk for psychosis. Further longitudinal studies are needed to explore the causal mechanisms underlying this relationship and to inform targeted prevention strategies.

**Disclosure of Interest:**

None Declared

